# Cognitive Function Changes in Older People. Results of Second Wave of Cognition of Older People, Education, Recreational Activities, NutritIon, Comorbidities, fUnctional Capacity Studies (COPERNICUS)

**DOI:** 10.3389/fnagi.2021.653570

**Published:** 2021-05-06

**Authors:** Sławomir Kujawski, Agnieszka Kujawska, Radosław Perkowski, Joanna Androsiuk-Perkowska, Weronika Hajec, Małgorzata Kwiatkowska, Natalia Skierkowska, Jakub Husejko, Daria Bieniek, Julia L. Newton, Karl J. Morten, Paweł Zalewski, Kornelia Kędziora-Kornatowska

**Affiliations:** ^1^Department of Hygiene, Epidemiology, Ergonomics and Postgraduate Training, Division of Ergonomics and Exercise Physiology, Collegium Medicum in Bydgoszcz, Nicolaus Copernicus University in Torun, Bydgoszcz, Poland; ^2^Department of Geriatrics, Collegium Medicum in Bydgoszcz, Nicolaus Copernicus University in Toruń, Toruń, Poland; ^3^Department of Physiology, Collegium Medicum in Bydgoszcz, Nicolaus Copernicus University in Toruń, Toruń, Poland; ^4^Department of Gastroenterology and Nutrition Disorders, Collegium Medicum in Bydgoszcz, Nicolaus Copernicus University in Toruń, Toruń, Poland; ^5^Population Health Sciences Institute, The Medical School, Newcastle University, Newcastle upon Tyne, United Kingdom; ^6^Nuffield Department of Women’s and Reproductive Health, The Women Centre, University of Oxford, Oxford, United Kingdom

**Keywords:** MMSE, MoCA, TMT B, occupational status, physical performance, cognitive reserve

## Abstract

**Background:**

Cognitive reserve explains why subjects with more years of education, professional achievement, or participation in recreational activities show less cognitive decline with aging. We hypothesize that levels of recreational travel, education, occupation, systemic health, physical performance, and current cognitive activity levels affect the trajectory of cognitive function in older, healthy people in Poland.

**Materials and Methods:**

Healthy, older people (*N* = 205) were examined and followed-up at 2 years. Participants completed physical and cognitive function assessments: including the Mini-Mental State Examination (MMSE), Montreal Cognitive Assessment (MoCA) and its two subtests Delayed Recall (DR) and Verbal Fluency (VF), and Trail Making Test Part B (TMT B). Factors associated with cognitive functioning were also examined.

**Results:**

The MMSE result significantly decreased over 2 years. No significant decrease in other cognitive tests was noted. However, the trajectory of cognitive tests results varied between individual participants. Percentage of variance of change was explained by the following predictors: 21 in MMSE, 24 in MoCA, 8 in DR, 25 in VF, and 24 in TMT B. Age and the presence of varicose veins were significantly linked to negative changes in MMSE and MoCA scores, while working in a professional occupational status associated with a higher score. The subgroup with varicose veins did worse on the Delayed Recall subtest of MoCA.

**Conclusion:**

Cognitive reserve could be extended by proxies of reserve that are related to systemic health and travel activity. The latter is a combination of social, physical, and cognitive activity and potentially might serve as an intervention to improve cognitive function in older people. However, due to the limitations of this study, results should be interpreted with caution and needs to be replicated in the further studies.

## Introduction

Aging is an unavoidable process in all living organisms. Discrepancies between biological and recorded age are often observed (Mianowany et al., 2004). Biological age, linked to body functionality is associated with how subjects adapt to the aging process and is a better indication of age than chronological age. In relation to the desire to successfully age, there is a growing interest in the promotion of health and prevention of diseases of the elderly (Schulz and Heckhausen, 1996). The concept of “cognitive reserve” described by Yaakov Stern in 2002 relates to the possibility of increasing the resistance of the brain to cognitive decline. The cognitive reserve hypothesis assumes that individual differences in cognitive performance are associated with biological differences in the brain and how neurons are coping with the aging process and is linked to the depletion of cognitive resources (Stern, 2002; Jodzio, 2012). Cognitive reserve explains why subjects with more years of education, professional achievement, or participation in recreational activities show less cognitive function decline during aging (Tucker and Stern, 2011).

Low gait velocity is related to poor cognitive function in older people (Peel et al., 2019). Moreover, physical activity might improve brain functioning (Pedersen, 2019). In addition, cognitive activity level and social activity correlated positively with cognitive function in healthy older adults (Clare et al., 2017; Kelly et al., 2017). Traveling abroad is a combination of social, physical, and cognitive activity. Bauer distinguishes activities related to trips such as sightseeing, physical activity, and interaction with nature, memory training, training of the senses of hearing and touching, attention and senses training, and taking part in additional language courses (Bauer, 2012).

Factors related to systemic health, in addition to lifestyle factor, also could be related to cognitive function level in older people. Visceral fat level was associated with mild cognitive impairment (MCI) in community-dwelling older Japanese women (Chiba et al., 2020). Hormones produced by visceral fat might play a role in cognitive function decline (Nota et al., 2020). Varicose veins presence is a risk factor for cardiovascular events and mortality (Wu et al., 2020). Varicose veins might induce inflammation (Markovic and Shortell, 2013) and endothelial dysfunction (Komarów et al., 2014). As a consequence, systemic inflammation and endothelial dysfunction could be related to cognitive function disturbance (Buie et al., 2019).

In this study, we hypothesize that levels of recreational travel, educational achievements, occupation, presence of varicose veins, visceral fat level, physical performance, and current cognitive activity levels could affect the trajectory of cognitive function in older, healthy people in Poland.

## Materials and Methods

### Study Group

#### Sample Size Calculation

Sample size calculation was done using the General Linear Mixed Model Power and Sample Size 3.0 calculator (GLIMMPSE 3.0) (Kreidler et al., 2013). Primary outcomes were MMSE and MoCA score changes in 2 years. Decrease in mean values of MMSE from 28 to 27.65 (SD = 3.5) and MoCA from 24 to 23.6 points (SD = 4.5) for outcomes were assumed. Using Hotelling Lawley Trace with power 0.8 and type I error rate 0.05, total sample size was calculated as 364. Assuming a 12% dropout rate, an additional 43 subjects were included, giving 407 participants in total.

#### Study Recruitment

**FIGURE 1:**
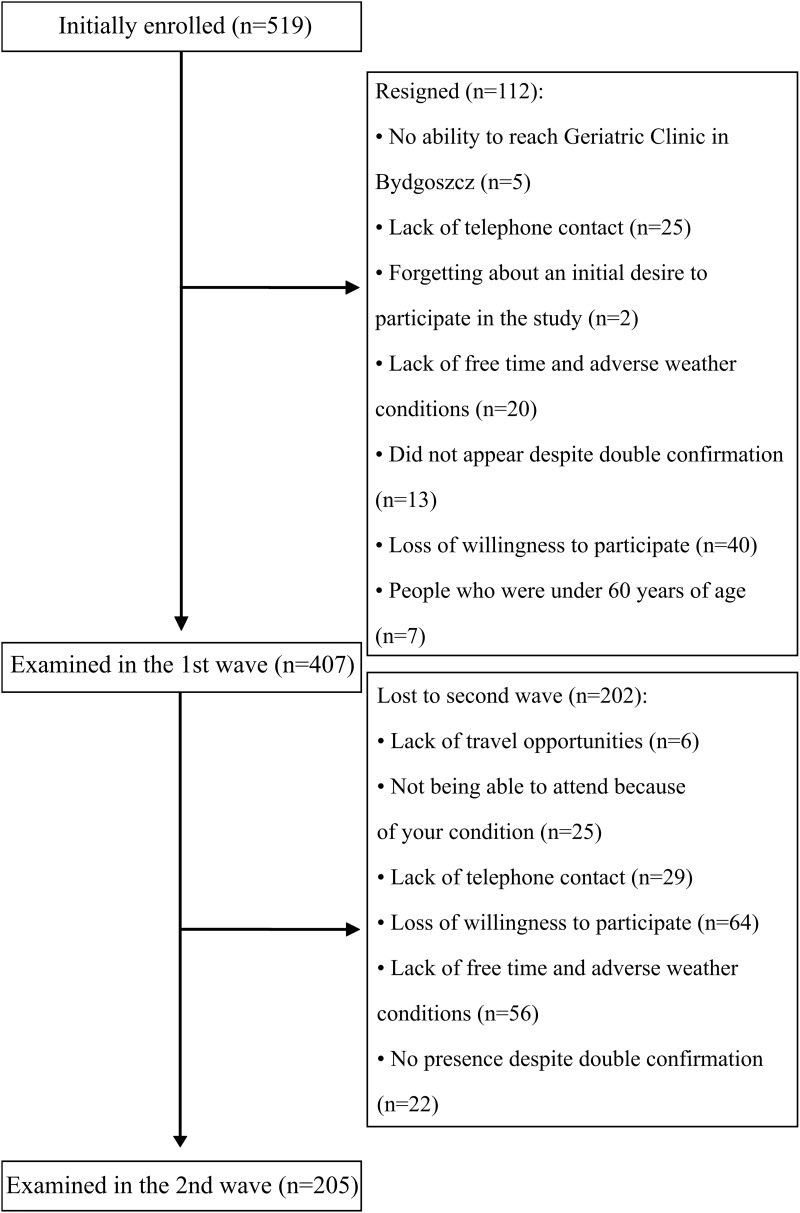
Flow chart of the study.

Study recruitment is summarized in Figure 1. In total, 407 participants (95 males) took part in the first series of assessments with 205 returning 2 years later for a second assessment (40 males).

Participants were recruited into studies based on a regional TV and radio advertising campaign, during health-promoting lectures at Collegium Medicum University and many older people’s organizations in Bydgoszcz, in Day Care Centers for the Elderly, and at various meeting groups for older people. Study marketing material included literature and information describing an opportunity to participate free-of-charge in a study measuring physical, physiotherapeutic, dietary, social, and cognitive measures for people 60 years old and over. Age under 60 years old was the only excluding factor from participation in the study. Examination was conducted in the Department of Geriatrics, Collegium Medicum University Hospital in Bydgoszcz, Poland. The study was approved by the Ethics Committee, Ludwik Rydygier Memorial Collegium Medicum in Bydgoszcz, Nicolaus Copernicus University, Torun (KB 340/2015). Written, informed consent was obtained from all participants.

### Assessment Methods

#### Cognitive Tests

Neuropsychological tests were conducted by two trained staff. The majority of the neuropsychological tests (97.1%) were conducted by the same person (SK). Cognitive function was examined using the Mini–Mental State Examination (MMSE), Montreal Cognitive Assessment (MoCA), and Trail Making Test Part B (TMT B). MMSE is a screening 30-point questionnaire, consisting of questions associated with current time and place, immediate recall and short-term verbal memory, calculation, language, and ability to construct a 3D figure (Folstein et al., 1983). A high score indicates better cognitive performance.

MoCA measures all main cognitive domains: visuospatial skills, short-term memory recall, executive functioning, phonemic fluency, and a two-item verbal abstraction (Nasreddine et al., 2005). During the MoCA test, the results of two subtests [Verbal Fluency (VF) subtest and Delayed Recall (DR)] of five nouns were considered separately during the analysis. The VF subtest result is the number of words in Polish starting with letter “S,” which are not own nouns (conjugation was prohibited).

The Trail Making Test part B is a rapidly assessed neuropsychological tool measuring various skills including executive functioning domain: visuospatial skills, task switching, and working memory (Reitan, 1958).

#### Functional Performance Assessment

An 8-ft up-and-go, where the subject is asked to get up from chair, walk an 8-ft distance to and around a marker placed on the floor, get back, and sit on the chair again, as fast as possible, is an indicator of functional performance, gait speed, and balance in a dynamic manner (Rikli and Jones, 1999).

#### Body Composition Analysis

Weight and body fat was measured using a Tanita BC-545 body-fat analyzer. Body fat was measured using bioelectric impedance analysis (BIA) made while subjects were standing on electrodes on the sole of the foot and holding electrodes in the hands. Weighing accuracy is 0.1%. Participants were weighed in light clothing. Respondents themselves gave information about height to reduce the amount of time spent on examination, and BMI was calculated in accordance with the WHO recommendations.

#### Occupation Status, Activity Level Questionnaire, and Physical Examination

Subjects were asked about time spent in education. Occupational status was initially assigned as: white collar worker, white collar worker in a managerial position, owner of the craft/entrepreneur, military/policeman/other uniformed services, seller/employee of trade, farmer in an individual farm, physical worker—qualified, unskilled worker; the last three were then recoded as “low occupational status” and the rest as “high occupational status.” Eventually, a binary variable for the highest occupational status during lifetime was generated (Table 1).

**TABLE 1:** Occupational status categories.

Initially encoding	Binary value
White collar worker	High occupational status
White collar worker in a managerial position	
Owner of the craft/entrepreneur	
Military/policeman/other uniformed services	
Seller/employee of trade	
Farmer in an individual farm	Low occupational status
Physical worker—qualified	
Unskilled worker	

Assessment of recreational and mental activities completed over the last year were assessed: reading newspapers and books, watching TV, listening to the radio, going to the café or restaurant, going to the cinema or theater, going to the concert, going to church, visiting friends or family, taking part in social group meetings, computer use, card game, chess/checkers, and solving crosswords. Answers on questions about frequency of current mental activities were coded in the following way: “never” was coded as 0, “once a year” as 1, “several times a year” as 2, “1–2 times a month” as 3, “once a week” as 4, “few times a week” as 5, “daily” as 6. The maximum score to obtain with this scale was 78 points (13 questions with 6 as the highest score in each item).

Traveling abroad in the last 3 years was coded in the following binary way: “no” as “0” and “yes” as “1.”

Subjects were asked about the presence of varicose veins on the lower limbs. Moreover, every subject underwent physical examination by trained staff, where presence of varicose veins was examined. Based on the results of physical examination, a binary variable was created with codes “0” (no) or “1” (yes).

#### Statistical Analysis

All statistical analyses were performed using the statistical package R (R Core Team, 2013). If assumption was met, dependent *t*-test was used for analysis, otherwise the Wilcoxon test was used to compare results of tests before vs after 2 years. To compare participants who were lost to follow-up vs those who were re-examined, independent *t*-test was used if assumptions on normality and homogeneity of variance were met, otherwise the Mann–Whitney U test was used. Mean and standard deviation (SD) values are presented.

The effect size for the Wilcoxon test was calculated using the following formula (Field, 2009):

$$r\;\frac{Z}{\sqrt{N}}$$

in which Z is the result of the Wilcoxon test, while N is the number of all observations. To calculate the effect size for the dependent *t*-test, the following r was used (Field, 2009):

$$r\;\sqrt{\frac{t^{2}}{t^{2}+d\,f}}$$

in which t is the result from the *t*-test, and df is the degrees of freedom.

To assess the impact of selected factors on the dynamics of cognitive functioning (before vs after 2 years), a linear mixed model with restricted maximum likelihood approach and *t* tests using the Satterthwaite method using R statistical packages (Lme4 and LmerTest) (Bates et al., 2015) were used. The subject and time factor were determined as random effects, and the factors explaining the change in cognitive functioning were determined as fixed effects. To calculate the confidence interval (95%) for the coefficient parameters, the confint command was used to calculate 5,000 simulations using the bootstrap method. The R2 value and confidence interval (–95%; 95%) were calculated using the r2beta command. The Dwplot command was used to visualize the results of the linear mixed models.

Violin plots were made using the ggstatsplot package (Patil, 2018). Red dots connected by a red line indicate the arithmetic mean value; a horizontal black line inside the frame means the median value. The green dots before and the orange dots after 2 years connected by dashed lines indicate the results of individual participants. The shape of the graph indicates the distribution of values. In circular histograms, the height of the column indicates the number of values, and the percentage of the value appears next to the value label.

## Results

**FIGURE 2:**
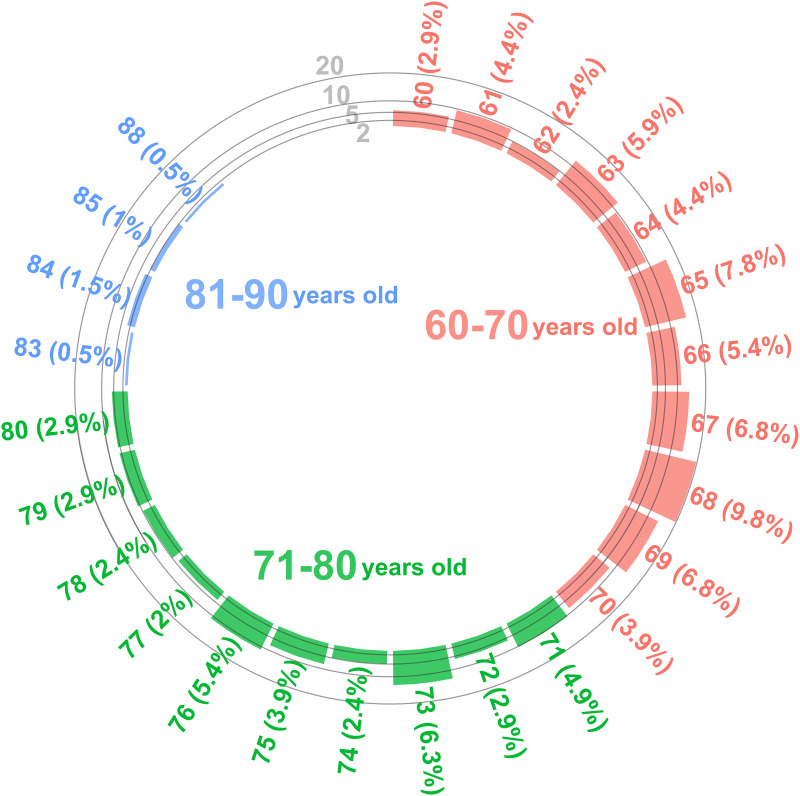
Histogram of age.

The mean age of the participants coming back for the 2-year follow-up assessment was 69.67 (–95% CI = 68.85; 95% CI = 70.5, range 60–88) with a breakdown of the different age groups shown in Figure 2.

Table 2 presents the distribution of education level of the re-examined group. Most of the participants (84.39%) obtained full secondary education or higher.

**TABLE 2:** Distribution of education categories in the re-examined group.

Category	Count	Percent
Primary education (incomplete)	1	0.49
Primary education	3	1.46
Vocational school education	22	10.73
Secondary education (incomplete)	6	2.93
Secondary education	92	44.88
higher Professional/engineering education	26	12.68
Master’s degree	52	25.37
PhD degree and higher	3	1.46

Table 3 presents the descriptive statistics of quantitative predictors included into the model used in explaining the variance of changes in cognitive test scores after 2 years. Data on years of education, 8-ft test, and visceral fat level from the initial time point were included into the linear mixed models. On the contrary, data on cognitive activity levels from both time points were presented further into models.

**TABLE 3:** Quantitative variables included in the linear mixed model.

Variables	Mean ± SD
Years of education (years)	14.37 ± 3.4
8-ft test (s)	6.02 ± 2.0
Visceral fat (level)	10.62 ± 3.4
Cognitive activity level 1st wave (points)	42.72 ± 8.2
Cognitive activity level 2nd wave (points)	41.94 ± 7.9

Table 4 shows the prevalence of varicose veins on the lower limbs. Data on sex, occupational status, and traveling abroad during the last 3 years at initial assessment were included into the linear mixed models.

**TABLE 4:** Qualitative variables included in the linear mixed model.

Parameter	Level of estimate	Number	Percentage
Sex	Male	40	19.5
	Female	165	80.5
Varicose veins on lower limbs before	Present	57	20.8
	Non-present	148	79.2
Varicose veins on lower limbs after 2 years	Present	74	36.3
	Non-present	130	63.7
Occupational status	High	173	86.07
	Low	28	13.93
Traveling abroad	Yes	122	59.5
	No	83	40.5

The MMSE score decreased significantly (27.8 ± 2.1 before vs 27.45 ± 2.3 after, *Z* = 2.56, *p* = 0.01, *r* = 0.15) (Supplementary Figure 1). No significant changes in MoCA score were noted (23.63 ± 3.5 before vs 23.21 ± 4.1 after, *Z* = 1.55, *p* = 0.12, *r* = 0.08) (Supplementary Figure 2). No significant changes in delayed recall subtest of MoCA were noted (2.25 ± 1.6 words recalled before vs 2.25 ± 1.7 after, *Z* = 0.07, *p* = 0.94, *r* = 0) (Supplementary Figure 3). No significant changes in verbal fluency subtest of MoCA was noted (12.73 ± 4.5 words spoken before vs 13.31 ± 4.8 after, *t* = –1.93, *p* = 0.06, *r* = 0.02) (Supplementary Figure 4). No significant changes in TMT B result were noted (138.36 ± 88.5 s before vs 132.88 ± 89.1 after, *Z* = 1.48, *p* = 0.14, *r* = 0.07) (Supplementary Figure 5).

**FIGURE 3:**
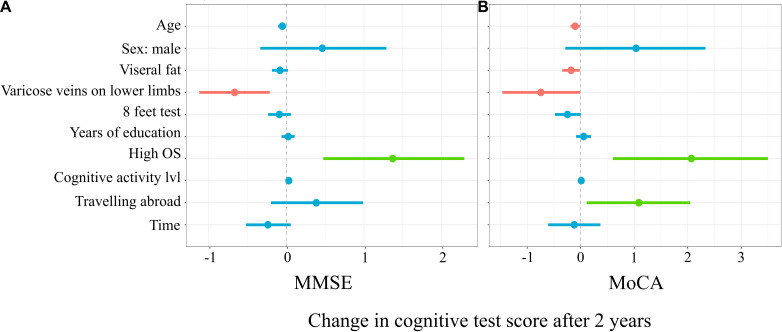
Mixed linear model predicting Mini-Mental State Examination (MMSE) **(A)** and Montreal Cognitive Assessment (MoCA) **(B)** score changes.

Figure 3A represents the results of the mixed linear model with MMSE as a predicted variable. The model explained 22% (17%; 31%) of the variance of the change in the MMSE score before vs after 2 years. For every additional year of age, there was0.05 (–0.1; 0.01) less point in the MMSE score (*t* = –1.99, *p* = 0.048). The subgroup with varicose veins on the lower limbs had a mean of 0.67 (–1.12; –0.21) points less in MMSE after 2 years (*t* = –2.99, *p* = 0.003). The subgroup with high occupational status was characterized by 1.37 (0.47; 2.3) more points in MMSE after 2 years (*t* = 3.09, *p* = 0.002). For every additional point in the cognitive activity level, there was 0.03 (0.0004; 0.06) more point in the MMSE score (*t* = 2.08, *p* = 0.04).

Figure 3B represents a linear mixed model on the results of MoCA. The applied model explained 24% (19%; 33%) of the variance in MoCA score changes. For every additional year of age, there was 0.1 (–0.19; –0.01) less point in the MoCA score (*t* = –2.45, *p* = 0.02). For every additional unit of visceral fat, there was 0.18 (–0.35; –0.01) less point in the MoCA score (*t* = –2.21, *p* = 0.03). The subgroup with varicose veins on the lower limbs had a mean of 0.74 (–1.46; –0.01) less point in MoCA after 2 years (*t* = –2.02, *p* = 0.04). For every second longer to finish the 8-ft test, in the first time point, there was 0.25 (–0.48; –0.002) point less in MoCA after 2 years (*t* = –2.1, *p* = 0.04). Participants with high occupational status and who traveled abroad in the 3-year period before the first time point had on a mean of 2.07 (0.6; 3.5) and 1.09 (0.11; 2.1), respectively, more points in MoCA in the second time point (*t* = 2.85, *p* = 0.005 and *t* = 2.31, *p* = 0.02.

The horizontal axis denotes the difference in the cognitive score test. The vertical dashed line indicates no change in cognitive test score (0). On the left side of the dashed line, there are predictors with a negative estimate, which are related to worse cognitive scores after 2 years, while on the right side of the dashed line, predictors related to better scores after 2 years are placed. Red lines indicate predictors, in which confidence interval values are negative, blue lines indicate predictors, in which confidence interval values cross zero, green lines indicate predictors, in which confidence interval values are positive.

**FIGURE 4:**
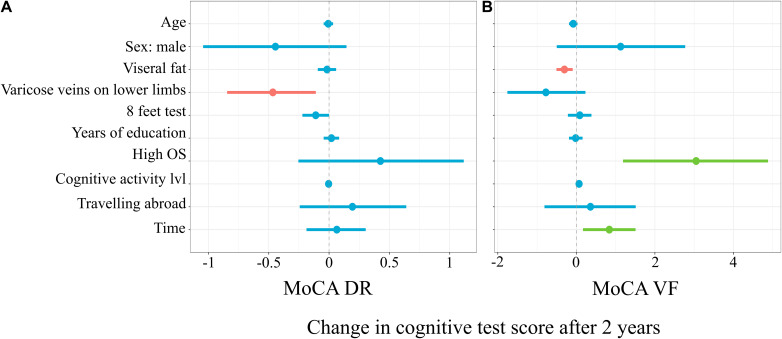
Mixed linear model predicting MoCA Delayed Recall **(A)** and Verbal Fluency **(B)** subtests score changes.

Figure 4A represents the linear mixed model on the results of the delayed recall subtests of the MoCA, which explained 9% (6%; 17%) of the variance in the score changes before vs after 2 years. Subjects with varicose veins on lower limb recalled a mean of 0.47 (–0.84; –0.11) less words in the MoCA delayed recall after 2 years (*t* = –2.56, *p* = 0.01). For every second longer to finish the 8-ft test, in the first time point, there was 0.11 (–0.22; 0.0005) point less words recalled after 2 years (*t* = –1.99, *p* = 0.048).

Figure 4B represents the linear mixed model on the results of verbal fluency subtests of MoCA, which explained 16% (12%; 25%) of the variance in the score changes. For every increase in the level of visceral fat, there was 0.3 (–0.51; –0.09) less words recalled after 2 years (*t* = –2.88, *p* = 0.004). The subgroup with a high occupational status had 3.05 (1.19; 4.88) more words pronounced in the verbal fluency subtest after 2 years (*t* = 3.26, *p* = 0.001). For every additional point in the cognitive activity level, there was 0.07 (0.0004; 0.14) more words pronounced in the verbal fluency subtest after 2 years (*t* = 2.11, *p* = 0.04).

The horizontal axis denotes difference in cognitive score test. The vertical dashed line indicates no change in cognitive test score (0). On the left side of the dashed line, there are predictors with negative estimate, which are related to worse cognitive scores after 2 years, while on the right side of the dashed line, predictors related to better scores after 2 years are placed. Red lines indicate predictors, in which confidence interval values are negative, blue lines indicate predictors, in which confidence interval values cross zero, green lines indicate predictors, in which confidence interval values are positive.

**FIGURE 5:**
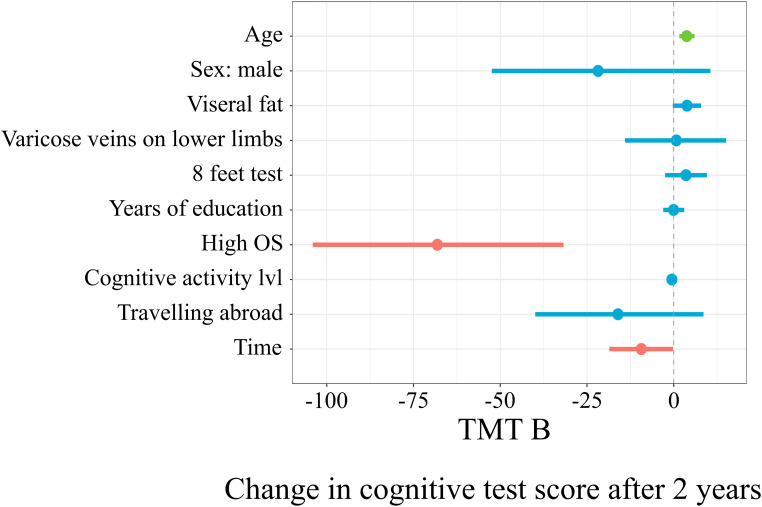
Mixed linear model predicting TMT B score changes.

Figure 5 represents the linear mixed model on the results of TMT B, which explained 25% (19%; 33%) of the score changes. With every additional year of age, there was an additional 3.74 (1.61; 6.01) s to complete TMT B after 2 years (*t* = 3.55, *p* = 0.0005). The subgroup with a high occupational status had, on average, 68.14 (–104.1; –31.77) less seconds to complete the test after 2 years (*t* = –3.74, *p* = 0.0002).

The horizontal axis denotes a difference in cognitive score test. The vertical dashed line indicates no change in cognitive test score (0). On the left side of the dashed line, there are predictors with a negative estimate, which are related to worse cognitive scores after 2 years, while on the right side of the dashed line, predictors related to better scores after 2 years are placed. Red lines indicate predictors, in which confidence interval values are negative, blue lines indicate predictors, in which confidence interval values cross zero, green lines indicate predictors, in which confidence interval values are positive.

Table 5 summarizes the comparison of cognitive function test subtest results before vs after 2 years.

**TABLE 5:** Comparison cognitive function before vs after 2 years.

Variable	Mean ± SD before	Mean ± SD after 2 years	Z (or t)	p	r
MMSE	27.8 ± 2.1	27.45 ± 2.3	2.56	0.01	0.15
MMSE Orientation	9.82 ± 0.6	9.77 ± 0.7	1.24	0.22	0.14
MMSE Memory	2.98 ± 0.2	2.98 ± 0.2	0.13	0.89	0.04
MMSE Attention	4.04 ± 1.3	4.04 ± 1.3	0.05	0.96	0.00
MMSE DR	2.71 ± 0.6	2.64 ± 0.6	1.24	0.21	0.11
MMSE Language	2.90 ± 0.3	2.89 ± 0.3	0.20	0.84	0.03
MMSE Ex. F.	3.95 ± 0.3	3.95 ± 0.3	0.17	0.86	0.03
MMSE 7. Writing	0.96 ± 0.2	0.96 ± 0.2	0.00	1.00	0.00
MMSE 8. Praxis	0.44 ± 0.5	0.21 ± 0.4	4.44	0.00001	0.33
MoCA	23.63 ± 3.5	23.21 ± 4.1	1.55	0.12	0.08
MoCA Visuospatial	3.7 ± 1.1	3.4 ± 1.2	2.96	0.003	0.18
MoCA Naming	2.89 ± 0.4	2.87 ± 0.4	0.76	0.44	0.12
MoCA Attention	4.8 ± 1.3	4.8 ± 1.3	0.04	0.97	0.00
MoCA Language	2.13 ± 1	2.17 ± 0.9	0.55	0.58	0.04
MoCA Abstraction	1.65 ± 0.6	1.56 ± 0.6	1.62	0.11	0.13
MoCA DR	2.25 ± 1.6	2.25 ± 1.7	0.07	0.94	0.00
DR- category cue	1.69 ± 1.1	1.50 ± 1.2	1.15	0.25	0.07
DR- choice cue	1.57 ± 0.9	1.83 ± 1.1	2.20	0.03	0.22
DR in overall	4.76 ± 0.5	4.68 ± 0.8	1.08	0.28	0.10
MoCA VF	12.73 ± 4.5	13.31 ± 4.8	T = –1.93	0.06	0.02
TMT B	136.9 ± 88.5	133.18 ± 89.1	1.48	0.14	0.07

## Discussion

In this study, a significant deterioration in MMSE results were observed over the 2-year study period. This was unexpected as previous studies have shown that MMSE is not an effective tool for monitoring cognitive decline in aging (Gluhm et al., 2013). Other cognitive function tests as MoCA and verbal fluency and delayed recall subtests and TMT B failed to detect a decline in cognitive function. As the vocabulary is part of the crystallized intelligence, this could potentially explain lack of decrease in the verbal fluency MoCA subtest (Craik and Bialystok, 2006). Moreover, lack of decrease in the rest test could be potentially explained by practice effect, as no alternative version of MMSE, MoCA, or TMT B were used in the second time point. Consistent with the results presented, in the 3-year Longitudinal Aging Study Amsterdam, deterioration of cognitive functioning was observed in all subjects (Comijs et al., 2004). In a review of the 2004 study by Bruscoli and Lovestone, they observed that the quantitative assessment of the conversion of people from MCI to dementia can vary between studies (Bruscoli and Lovestone, 2004). Potentially due to the practice effect, improvement in cognitive function was noted in some participants. In this study, we showed that the presence of varicose veins links to a greater decline in MMSE and MoCA scores over 2 years, while high occupational status was related to better results. The subgroup who actively traveled abroad for leisure had better functional fitness correlating to an improved MoCA score after 2 years. The subgroup with varicose veins did worse on the Delayed Recall subtest of MoCA. Visceral fat level was related to a decrease in verbal fluency result, while high occupational status was related to better results. Over the 2 years, age was related to worse results in the TMT B test, while high occupational status associated with better results.

### Sex, Education, Occupational Status, Cognitive Activity Level

In the above study, sex was not a significant predictor for change in any cognitive test, while high occupational status was a positive predictor of good scores in MMSE, MoCA tests, verbal fluency subtests, and TMT B scores were also higher. A higher level of cognitive activity level was related to better results in verbal fluency and global cognitive function measured by MMSE after 2 years. However, years spent in education was not a significant predictor of cognitive function scores after 2 years. Presumably, the lack of correlation between these factors could be explained by specific distribution of years of education in the examined group in the above study. Most of the participants (84.39%) obtained full secondary education or higher. Therefore, the above sample is underrepresented in subjects with a lower level of education than secondary. In the above study, an underrepresentation of males was observed (19.5%). Therefore, it might explain why sex was not a significant predictor in the above study, contrary to previous longitudinal studies, where higher resilience to cognitive decline was noted in older women compared with men (McCarrey et al., 2016). A meta-analysis study shows that occupational status and participation in cognitively stimulating activities is related to cognitive function in older people (Opdebeeck et al., 2016). Future studies should focus on determining why such interventions promote successful cognitive aging rather than focus on the time spent in education. What is interesting, in the present study, years of education were not related to change in cognitive function decline. In previous studies, it was suggested that IQ is a more powerful predictor of cognitive reserve than years spent in education (Alexander et al., 1997). Armstrong et al. (2012) has suggested that IQ may have more influence than education level in Parkinson’s Disease-Mild Cognitive Impairment. IQ level is strongly related to education attainment (Butler et al., 1985). In the above study, the IQ of the participants was not determined. It is interesting to consider why years of education were not related to cognitive function changes in our Polish cohort. One possible explanation is that many factors influence the ability of subjects to progress through the education system. Participants may not have the opportunity to attend higher education even though they have a high IQ. The highest occupational status and subject obtained throughout their life might be a more reliable indicator of IQ and, hence, explain the link with this group having a slower rate of cognitive function decline compared with the subjects with higher levels of education where a correlation was not observed. Authors of the Maastricht Aging Study (MAAS) underlie the role of intellectual abilities in comparison with developmental and socioeconomic characteristics of early life in functional decline and successful aging (Bosma et al., 2007). A low socioeconomic status (measured based on wealth) was related to greater decline in cognitive function during 6 to 8 years (Steptoe and Zaninotto, 2020). Moreover, alternative ways of explanation of relationship between IQ and job performance were described (Byington and Felps, 2010). The authors suggest that subjects with a higher IQ have an opportunity to gain access to developmental resources allowing them to undergo the process of on-the-job learning (Byington and Felps, 2010). In addition, subjects with a high occupational status will also have the added long-term cognitive stimulation of their work, which not everybody of a degree status will not necessary have. An active and challenged mind into old age is potentially the key to aging well. Further studies should examine the relationship between education level, occupational status, socioeconomic status, and IQ level with each other and with cognitive function changes during lifetime. Identification of modifiable factors that influence cognitive function dynamics would be of high importance for public health recommendations.

### Visceral Fat Level

In this study, levels of visceral fat level were a negative predictor of verbal fluency and global cognitive function measured by the MoCA test changes after 2 years. Previous studies have suggested that patterns of fat storage might play an important role in pathophysiological mechanisms of obesity and cognitive impairment (Papachristou et al., 2015). The link between obesity and reduced executive function might be related to fat stored in a specific region such as visceral fat (Schwartz et al., 2013). Significant, negative correlation was found between phonemic fluency and visceral fat in adolescents (12–18 years old) (Schwartz et al., 2013). Fat mass and obesity-associated gene (FTO) polymorphisms were associated with worse verbal fluency in overweight and obese older men (Benedict et al., 2011). Moreover, hormones produced by visceral fat might presumably cross the blood–brain barrier and play a role in cognitive function decline (Nota et al., 2020). Leptin and adiponectin can cross the blood–brain barrier (Lee et al., 2019). A negative correlation between the level of leptin in the peripheral blood with the volume of brain gray matter was observed (Pannacciulli et al., 2007). In contrast, a higher level of adiponectin seems to be associated with more beneficial cognitive function dynamics in older subjects (Rizzo et al., 2020). However, in the above study, levels of hormones produced by fat tissue were not determined. Therefore, the inability to explain the relationship between visceral fat level in cognitive function changes is a potential limitation of the above results.

The relationship between visceral fat and global cognition decline and brain changes have been observed. Debette et al. showed that middle-aged subjects had an inverse association between levels of abdominal, especially visceral, fat with total brain volume and low cognitive function scores (Debette et al., 2010; Isaac et al., 2011). In a study of older subjects, visceral fat was negatively correlated with the level of cognitive function in subjects under 70 years (Yoon et al., 2012).

Cortical thinning (gray matter volume) in young and middle-aged populations was linked to BMI and amounts of visceral adipose tissue in patients with diabetes (Veit et al., 2014). Various factors including aerobic fitness, blood pressure, and cognitive functioning strongly link to BMI (Waldstein and Katzel, 2006). Drawing conclusions from associations linked to BMI are challenging because BMI is not a reliable indicator of body composition, and more research is needed to untangle the multiple factors including lean body mass, aerobic fitness, physical activity, and blood pressure measurement associated with BMI.

### Fitness Level and Cognitive Decline

In this study, the 8-ft up-and-go test results were a significant predictor of changes in the MMSE test and delayed recall subtest. Among older people, executive functions are positively related to a 4-m course at usual pace and walking speed on a 7-m obstacle course at fast pace. The authors suggest that execution of a physical performance task requires attention resources (Ble et al., 2005). A meta-analysis shows that poor gait results predict dementia with gait issues also observed in diabetes when management of the disease starts to decline (Beauchet et al., 2016; Henderson et al., 2019). In dementia, the type of dementia is important; other than Alzheimer disease, poor gait performance is a stronger predictor of dementia (Henderson et al., 2019). A slight slowdown in gait and impairment of cognitive functions co-occur in the elderly (Atkinson et al., 2007). Correlations between specific cognitive functions and gait speed in the elderly in longitudinal studies are less pronounced than in cross-sectional studies (Callisaya et al., 2015). Participants in “The Tasmanian Study of Cognition and Gait,” aged 60–85, randomly selected from the electoral list, were evaluated twice in 3 years. A decrease in executive functions, but not other cognitive domains, was associated with a decrease in gait speed (Callisaya et al., 2015). The results of the study indicate both cross-sectional and longitudinal links between cognitive functioning and gait speed among older people living in the community. Worse verbal fluency and lower psychomotor speed were associated with lower maximum gait speed and greater deterioration of maximum gait speed over time (Soumaré et al., 2009).

### Varicose Veins

The presence of varicose veins was a strong negative predictor of MMSE, MoCA, and MoCA Delayed Recall subtest score changes over the 2-year study period. Varicose veins could be a sign of venous insufficiency. Several studies have proposed a correlation between venous insufficiency and cardiac disorders (Auzky et al., 2011). In addition, varicose vein presence might be related to peripheral artery disease, although some confounding factors may be involved (Chang et al., 2018). Other cross-sectional studies also indicate that peripheral arterial disease and cardiovascular disorders were associated with an increased risk of vascular dementia (Newman et al., 2005; Laurin et al., 2007). The presence of varicose veins is related to an increased risk of cardiovascular events and mortality (Wu et al., 2020). Varicose veins might induce inflammation (Markovic and Shortell, 2013) and endothelial dysfunction (Komarów et al., 2014). In turn, high body fat percentage, systemic inflammation, and endothelial dysfunction might be related to cognitive function impairment (Buie et al., 2019). Therefore, further studies should examine this indirect relationship between varicose veins and cognitive function in older people. If such a correlation would be confirmed, then varicose veins presence could serve as an easy-to-examine factor relevant to cognitive function in older people.

### Traveling Abroad

Traveling abroad in the last 3 years before the first time point was a significant predictor of changes in MoCA score after 2 years. Moreover, traveling abroad was a significant predictor of cognitive function in the baseline results in this study (Kujawski et al., 2018). However, these results need to be treated with caution as many factors could link to enhanced and reduced travel activity. Self-selection might occur, where participants who did not travel abroad may lack motivation, were depressed or fearful of traveling, had poor economic and/or health status, and had difficulty accessing information (Crawford and Godbey, 1987). Moreover, marital status and small size of social networks might be other restriction factors (Nyaupane and Andereck, 2008). Moreover, traveling abroad might be related to some level of fluency in the second language, and bilingualism might contribute to cognitive reserve (Arce Rentería et al., 2019). Travel activity might work as an aging intervention by influencing a range of parameters feeding into cognitive function improvement. Bauer distinguishes activities related to trips such as sightseeing, physical activity, and interaction with nature, memory training, training of the senses of hearing and touching, attention and sense training, and taking part in additional language courses (Bauer, 2012). To our best knowledge, the above study is the first to examine the relationship between traveling abroad and cognitive function changes in older people. However, a binary variable considering traveling abroad was included only. Therefore, further studies should use continuous variables to examine various types of touristic activities and its relation to changes in cognitive function.

### Study Limitations

Two screening tests were used in this study. Future longitudinal studies carried out in Poland should include in their methodology tests that assess all cognitive domains and are not based almost exclusively on the visual and basic motor functions. It is important that the next studies include a representative sample of the whole country if the results are to be relevant to the whole Polish population; only people from Bydgoszcz and the surrounding area were included in this study. Subject drop out in studies like this one can be higher in subjects with greater cognitive impairment or who are sick introducing potential bias. The authors of a systematic review of 12 articles that analyze the number of dropouts in longitudinal studies, other than death, showed that people who did not participate in the study for reasons other than death, were older and had greater cognitive impairment. Sick or weak people are more likely to give up, and people who cannot be contacted may have worse health than other respondents (Chatfield et al., 2005).

A potential limitation of the above results is the low number of males (19.5%) included. Moreover, the IQ of the participants were not examined. In addition, recruitment based mainly on advertisement in mass media is not free from limitations. In further studies on aging in Poland, recruitment methods should be carefully chosen to limit potential bias, representational fraction of males should be included, and IQ level of participants should be examined.

The limitation of these presented research results is the method of recruitment into the study *via* mass media, which could lead to bias. Moreover, the number of people who resigned from participating in the second wave could be considered as high (*n* = 202), leading the result to be potentially underpowered. In the MAAS, people who refused to follow the follow-up had a lower level of education and worse baseline results in cognitive tests. Those who did not survive by the time of re-examination were more often men, were older, and had worse results in cognitive tests than the other participants. The authors concluded that despite the fact that participants continuing the study and people who were dropouts differed in sociodemographic and cognitive characteristics, dropouts seemed to have little impact on the analysis of cognitive dynamics (Van Beijsterveldt et al., 2002).

## Conclusion

The MMSE results significantly decreased after 2 years. No significant decrease in other cognitive tests was noted. However, the trajectory of the cognitive tests results varied between individual participants.

Age and the presence of varicose veins associated with declining MMSE and MoCA scores over 2 years, while high occupational status was linked positively to a slower decline in test scores. Age was a negative predictor of TMT B, while a high occupational status was a positive one. Moreover, there was a relationship between travel activity and functional fitness and MoCA score changes and between functional fitness and MoCA Delayed Recall subtest score changes. Varicose vein presence was related to decline in the results of Delayed Recall subtest of MoCA within 2 years. Visceral fat level was a negative predictor of verbal fluency result changes, while high occupational status was a positive predictor. Moreover, visceral fat level was a negative predictor of MoCA score changes. Cognitive activity level was a positive predictor of changes in MMSE and verbal fluency.

Based on those results, we suggest that the original cognitive reserve hypothesis should be extended by proxies of reserve that are related to systemic health and travel activity. The latter is a combination of social, physical, and cognitive activity and potentially might serve as an intervention to improve cognitive function in older people. However, due to the limitations of this study, results should be interpreted with caution. Moreover, further studies on the relationship between the abovementioned factors with cognitive function in older people changes are needed.

## Data Availability Statement

The raw data supporting the conclusions of this article will be made available by the authors, without undue reservation.

## Ethics Statement

The studies involving human participants were reviewed and approved by Ethics Committee, Ludwik Rydygier Memorial Collegium Medicum in Bydgoszcz, Nicolaus Copernicus University, Torun. The patients/participants provided their written informed consent to participate in this study.

## Author Contributions

All authors listed have made a substantial, direct, and intellectual contribution to the work, and approved it for publication.

## Conflict of Interest

The authors declare that the research was conducted in the absence of any commercial or financial relationships that could be construed as a potential conflict of interest.
