# Supplementary material for: Cognitive Function Changes in Older People. Results of Second Wave of Cognition of Older People, Education, Recreational Activities, NutritIon, Comorbidities, fUnctional Capacity Studies (COPERNICUS)
Source: Front Aging Neurosci. 2021 May 6;13:653570. doi: 10.3389/fnagi.2021.653570 (PMC8134550; doi:10.3389/fnagi.2021.653570)
Supplement: Supplementary file 1 [file Table_1.DOCX]

Supplementary Material

# Supplementary Data

# Supplementary Figures and Tables


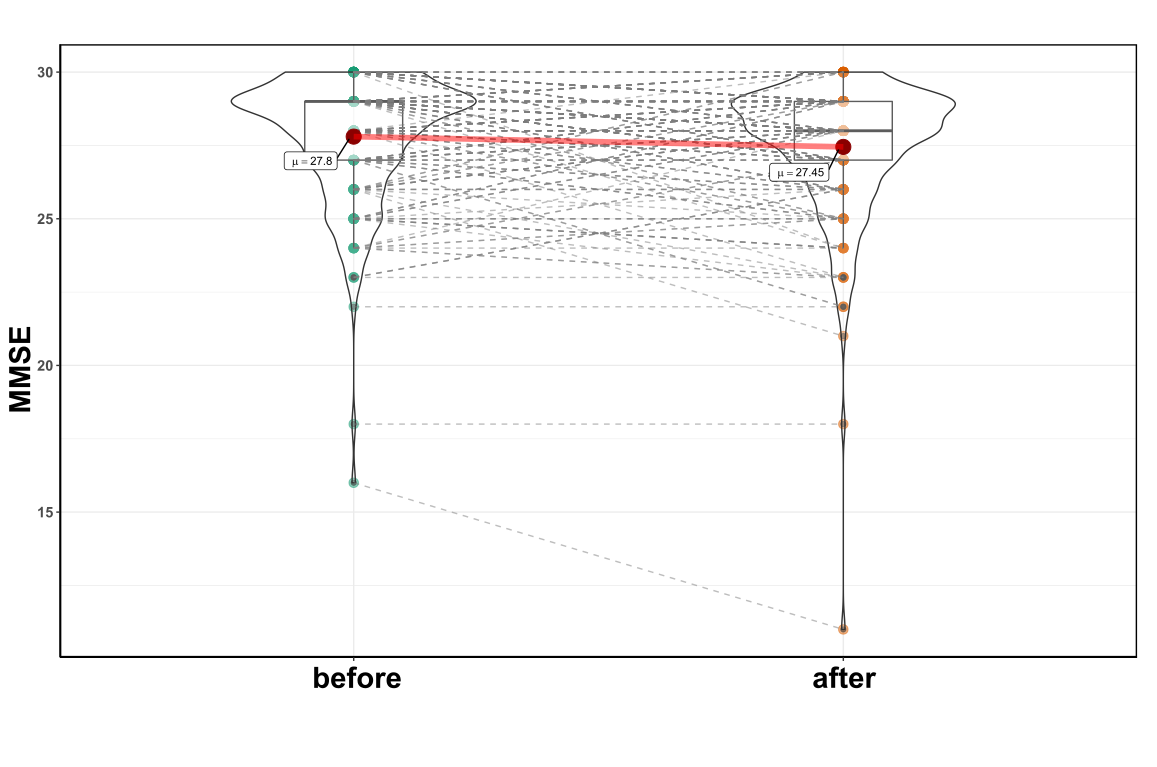


**Supplementary Figure 1. Change of MMSE score.** Before denotes initial time point, after denotes time point after 2 years

**
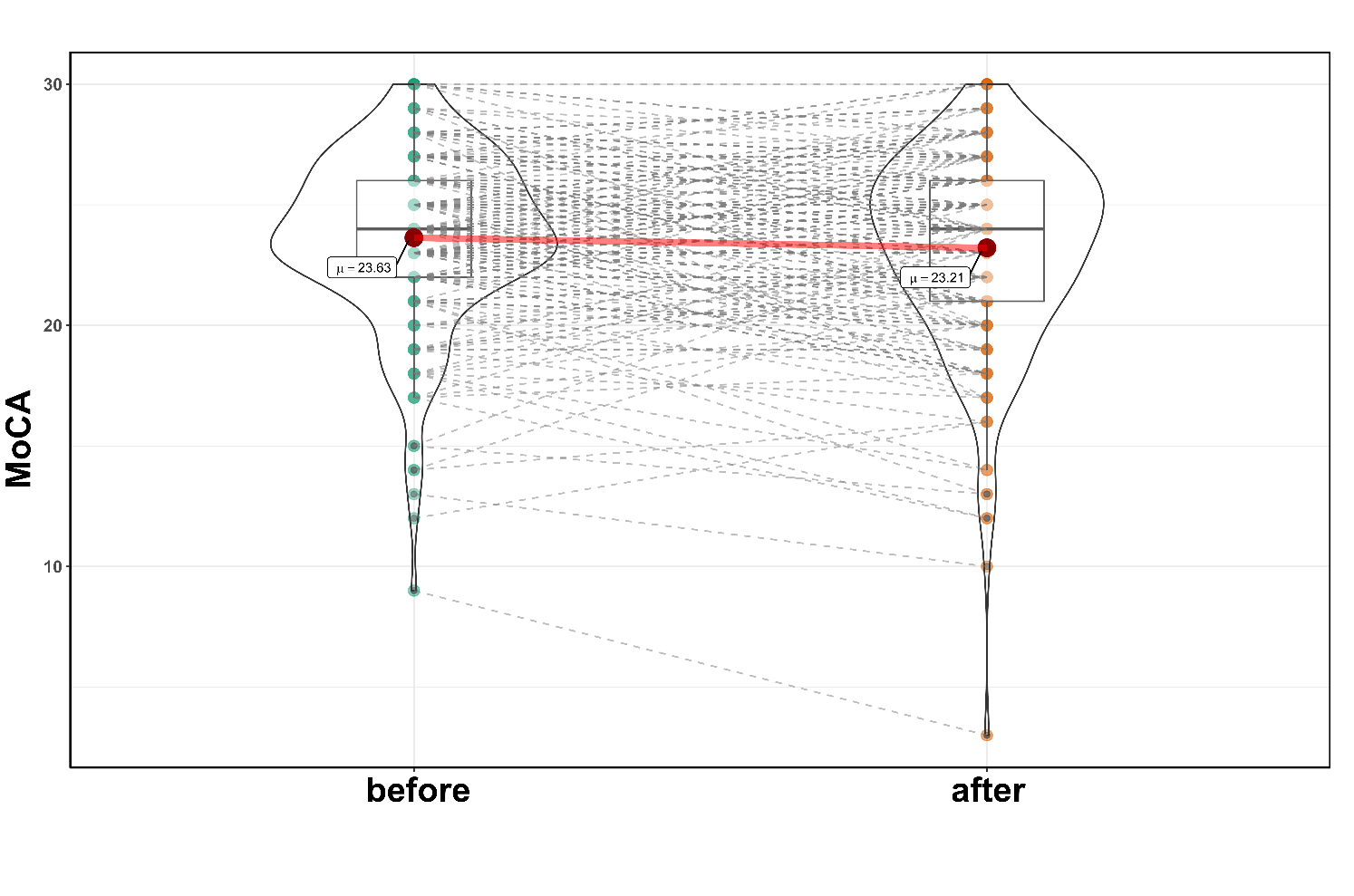
**

**Supplementary Figure 2. Change of MoCA score.** Before denotes initial time point, after denotes time point after 2 years

**
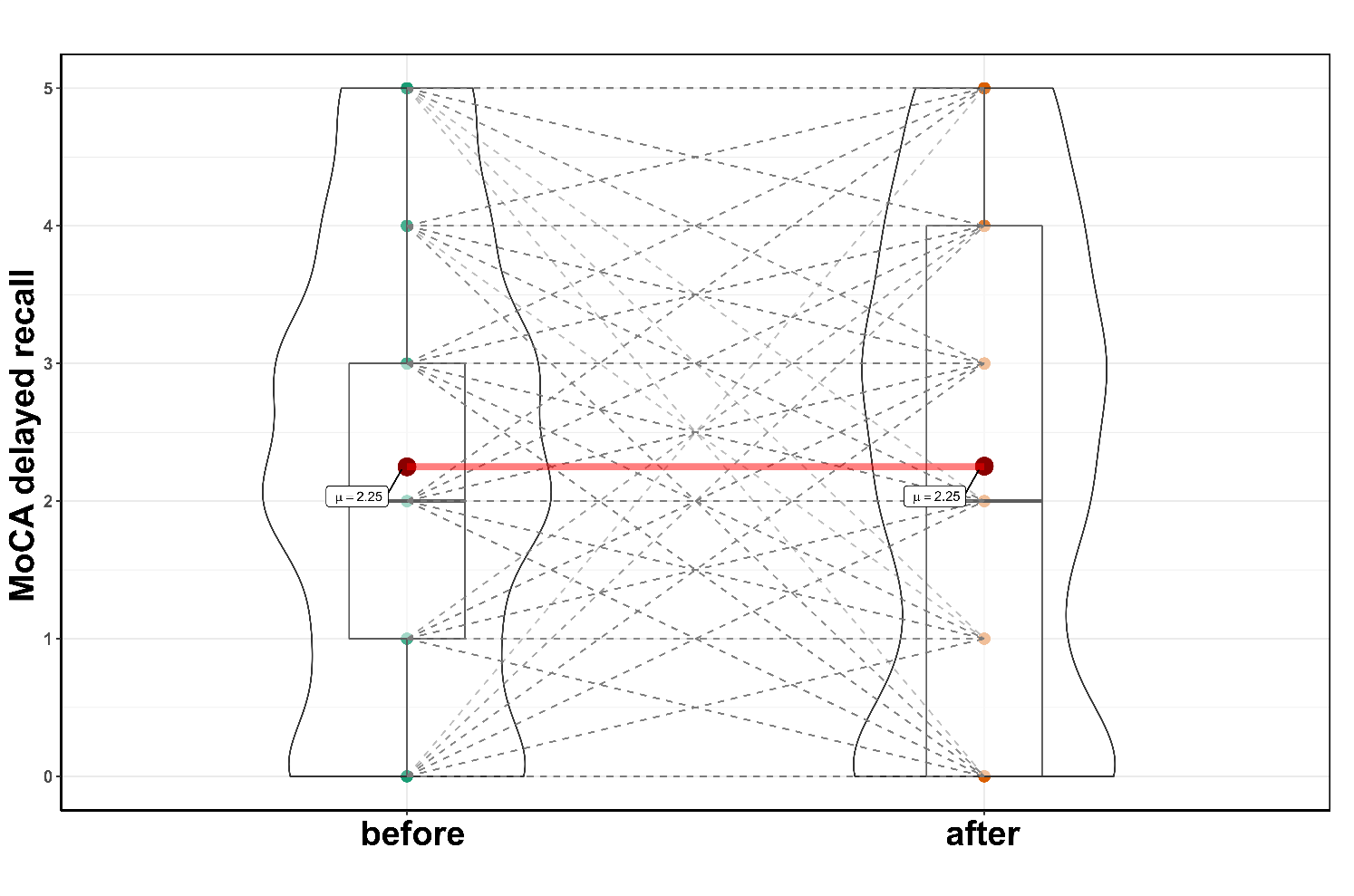
**

**Supplementary Figure 3. Change of MoCA delayed recall score.** Before denotes initial time point, after denotes time point after 2 years

**
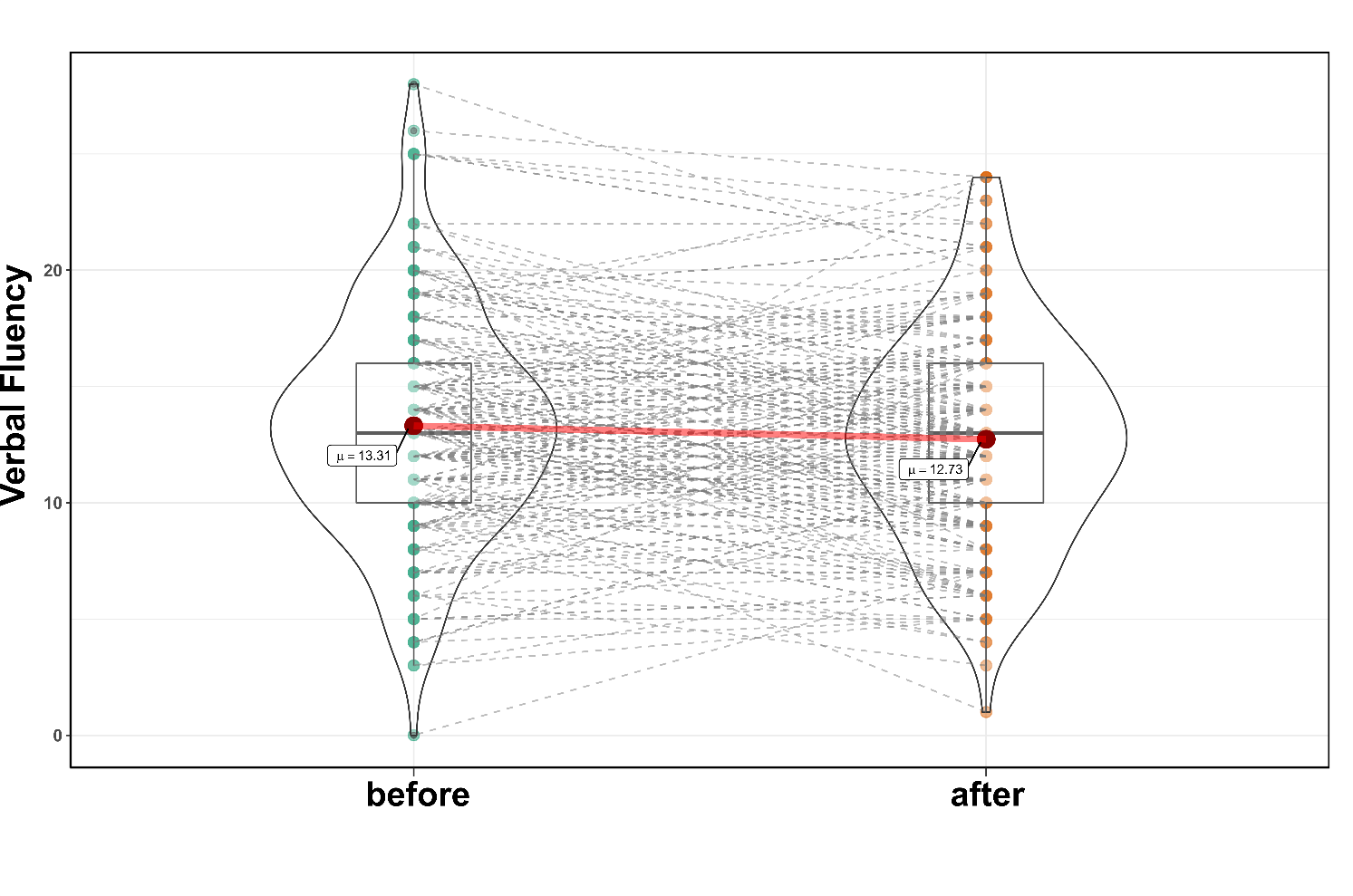
**

**Supplementary Figure 4. Change of MoCA Verbal Fluency score.** Before denotes initial time point, after denotes time point after 2 years


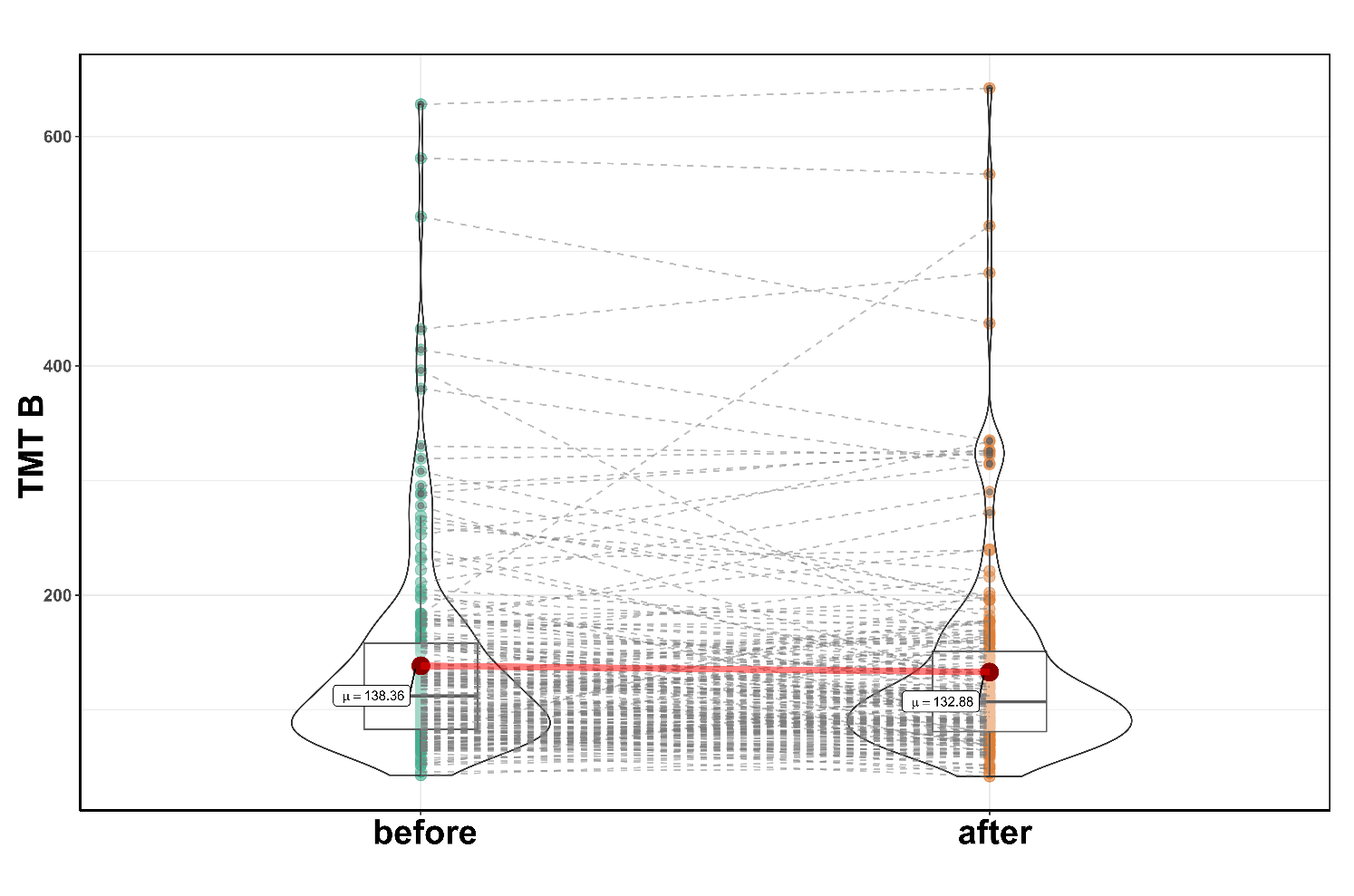


**Supplementary Figure 5. Changes of TMT B score.** Before denotes initial time point, after denotes time point after 2 years

**Supplementary table S1. Comparison of lost to follow-up vs re-examined subjects**

| **Variable** | **Not examined in the second wave mean±SD** | **Re-examined mean±SD** | **Z or t value** | **p** |
| --- | --- | --- | --- | --- |
| Age [years] | 69.84±6.3 | 69.66±6.0 | 0.01 | 0.99 |
| MMSE [points] | 27.33±2.4 | 27.80±2.1 | -2.24 | 0.03 |
| MoCA [points] | 22.74±3.7 | 23.63±3.5 | -2.46 | 0.01 |
| MoCA Delayed Recall [words] | 2.26±1.7 | 2.25±1.6 | 0.10 | 0.92 |
| MoCA Verbal Fluency [words] | 12.02±4.4 | 12.73±4.5 | t = -1.59 | 0.11 |
| TMT B [seconds] | 162.74±99.4 | 136.90±88.5 | 3.33 | <0.01 |
| Years of education [years] | 13.55±3.1 | 14.37±3.4 | -2.24 | 0.02 |
| Cognitive activity lvl [points] | 40.06±8.5 | 42.72±8.2 | -3.05 | <0.01 |

**Supplementary table S2. Mixed linear model predicting MMSE score changes**

| **Predictor** | **Estimate** | **CI** | **t value** | **p** |
| --- | --- | --- | --- | --- |
| Age [years] | -0.05 | -0.10; 0.001 | -1.99 | 0.048 |
| Sex [male] | 0.46 | -0.33; 1.29 | 1.18 | 0.24 |
| Visceral fat [units] | -0.08 | -0.18; 0.02 | -1.65 | 0.10 |
| Varicose veins on lower limbs [presence] | -0.67 | -1.12; -0.21 | -2.99 | 0.003 |
| 8 feet test [seconds] | -0.09 | -0.23; 0.06 | -1.25 | 0.21 |
| Years of education [years] | 0.03 | -0.06; 0.11 | 0.61 | 0.54 |
| High OS [presence] | 1.37 | 0.47; 2.29 | 3.09 | 0.002 |
| Cognitive activity lvl [score] | 0.03 | 0.0004; 0.06 | 2.08 | 0.04 |
| Travelled abroad [presence] | 0.39 | -0.20; 0.99 | 1.34 | 0.18 |
| time | -0.24 | -0.52; 0.06 | -1.58 | 0.12 |

**Supplementary table S3. Mixed linear model predicting MoCA score changes**

| **Predictor** | **Estimate** | **CI** | **t value** | **p** |
| --- | --- | --- | --- | --- |
| Age [years] | -0.10 | -0.19; -0.01 | -2.45 | 0.02 |
| Sex [male] | 1.03 | -0.29; 2.33 | 1.61 | 0.11 |
| Visceral fat [units] | -0.18 | -0.35; -0.01 | -2.21 | 0.03 |
| Varicose veins on lower limbs [presence] | -0.74 | -1.46; -0.01 | -2.02 | 0.04 |
| 8 feet test [seconds] | -0.25 | -0.48; 0.002 | -2.10 | 0.04 |
| Years of education [years] | 0.06 | -0.09; 0.19 | 0.82 | 0.41 |
| High OS [presence] | 2.07 | 0.60; 3.50 | 2.85 | 0.005 |
| Cognitive activity lvl [score] | 0.01 | -0.04; 0.06 | 0.40 | 0.69 |
| Travelled abroad [presence] | 1.09 | 0.11; 2.05 | 2.31 | 0.02 |
| time | -0.12 | -0.61; 0.37 | -0.48 | 0.63 |

**Supplementary table S4. Mixed linear model predicting MoCA Delayed Recall changes**

| **Predictor** | **Estimate** | **CI** | **t value** | **p** |
| --- | --- | --- | --- | --- |
| Age [years] | -0.01 | -0.04; 0.03 | -0.33 | 0.741 |
| Sex [male] | -0.44 | -1.04; 0.14 | -1.46 | 0.145 |
| Visceral fat [units] | -0.02 | -0.09; 0.06 | -0.43 | 0.667 |
| Varicose veins on lower limbs [presence] | -0.47 | -0.84; -0.11 | -2.56 | 0.011 |
| 8 feet test [seconds] | -0.11 | -0.22; 0.0005 | -1.99 | 0.048 |
| Years of education [years] | 0.02 | -0.04; 0.08 | 0.60 | 0.546 |
| High OS [presence] | 0.43 | -0.25; 1.12 | 1.24 | 0.217 |
| Cognitive activity lvl [score] | 0.00 | -0.03; 0.02 | -0.30 | 0.767 |
| Travelled abroad [presence] | 0.19 | -0.24; 0.64 | 0.87 | 0.383 |
| time | 0.06 | -0.19; 0.30 | 0.52 | 0.605 |

**Supplementary table S5. Mixed linear model predicting MoCA Verbal Fluency changes**

| **Predictor** | **Estimate** | **CI** | **t value** | **p** |
| --- | --- | --- | --- | --- |
| Age [years] | -0.08 | -0.19; 0.03 | -1.45 | 0.15 |
| Sex [male] | 1.13 | -0.50; 2.77 | 1.36 | 0.18 |
| Visceral fat [units] | -0.30 | -0.51; -0.09 | -2.88 | 0.004 |
| Varicose veins on lower limbs [presence] | -0.78 | -1.76; 0.23 | -1.55 | 0.12 |
| 8 feet test [seconds] | 0.09 | -0.21; 0.39 | 0.58 | 0.56 |
| Years of education [years] | -0.02 | -0.19; 0.16 | -0.20 | 0.84 |
| High OS [presence] | 3.05 | 1.19; 4.88 | 3.26 | 0.001 |
| Cognitive activity lvl [score] | 0.07 | 0.004; 0.14 | 2.11 | 0.04 |
| Travelled abroad [presence] | 0.36 | -0.81; 1.51 | 0.60 | 0.55 |
| time | 0.84 | 0.17; 1.51 | 2.45 | 0.02 |

**Supplementary table S6. Mixed linear model predicting TMT B changes**

| **Predictor** | **Estimate** | **CI** | **t value** | **p** |
| --- | --- | --- | --- | --- |
| Age [years] | 3.74 | 1.61; 6.01 | 3.55 | 0.0005 |
| Sex [male] | -21.79 | -52.45; 10.59 | -1.43 | 0.15 |
| Visceral fat [units] | 3.84 | -0.28; 7.91 | 1.89 | 0.06 |
| Varicose veins on lower limbs [presence] | 0.75 | -14.05; 15.09 | 0.10 | 0.92 |
| 8 feet test [seconds] | 3.53 | -2.47; 9.56 | 1.18 | 0.24 |
| Years of education [years] | -0.04 | -3.03; 3.04 | -0.03 | 0.98 |
| High OS [presence] | -68.14 | -104.05; -31.77 | -3.74 | 0.0002 |
| Cognitive activity lvl [score] | -0.54 | -1.66; 0.55 | -0.98 | 0.33 |
| Travelled abroad [presence] | -16.07 | -39.94; 8.58 | -1.34 | 0.18 |
| time | -9.33 | -18.56; -0.23 | -2.01 | 0.05 |
